# The Postencounter Form System: Viewpoint on Efficient Data Collection Within Electronic Health Records

**DOI:** 10.2196/17429

**Published:** 2020-04-06

**Authors:** Philip Held, Randy A Boley, Walter G Faig, John A O'Toole, Imran Desai, Alyson K Zalta, Jawad Khan, Shannon Sims, Michael B Brennan, Rebecca Van Horn, Angela C Glover, Bala N Hota, Brian D Patty, S Shafiq Rab, Mark H Pollack, Niranjan S Karnik

**Affiliations:** 1 Department of Psychiatry and Behavioral Sciences Rush University Medical Center Chicago, IL United States; 2 Research Institute Children's Hospital of Philadelphia Philadelphia, PA United States; 3 Department of Information Services Rush University Medical Center Chicago, IL United States; 4 Department of Psychological Science University of California, Irvine Irvine, CA United States; 5 Vizient, Inc Chicago, IL United States; 6 Department of Internal Medicine Rush University Medical Center Chicago, IL United States

**Keywords:** electronic health record, data collection, veterans

## Abstract

Electronic health records (EHRs) offer opportunities for research and improvements in patient care. However, challenges exist in using data from EHRs due to the volume of information existing within clinical notes, which can be labor intensive and costly to transform into usable data with existing strategies. This case report details the collaborative development and implementation of the postencounter form (PEF) system into the EHR at the Road Home Program at Rush University Medical Center in Chicago, IL to address these concerns with limited burden to clinical workflows. The PEF system proved to be an effective tool with over 98% of all clinical encounters including a completed PEF within 5 months of implementation. In addition, the system has generated over 325,188 unique, readily-accessible data points in under 4 years of use. The PEF system has since been deployed to other settings demonstrating that the system may have broader clinical utility.

## Introduction

### Background

The increasing use of electronic health records (EHR) across medical systems offers unique opportunities for research and quality improvement, and has the potential to significantly improve patient care [1]. Data captured in the EHR provides important insights about patients’ characteristics, treatment needs, visit frequencies, and other types of information. The voluminous amount of information contained in EHRs also enables the development of advanced models to predict the course of diseases, examine treatment effectiveness, and determine treatment options [2,3]. Despite the wealth of information contained in EHRs, a major challenge exists when it comes to extracting usable data to advance the delivery of care and clinical operations. This is largely due to extensive amounts of text-based information existing within clinical progress notes, which can be labor intensive and costly to transform into usable data points [4,5].

Manual chart reviews and data coding or more advanced analytic techniques such as natural language processing (NLP) are strategies that have been employed to make text-based information in EHRs more usable. However, reviewing charts and extracting data manually or training a machine learning model using NLP to identify the proper information requires significant time and are consequently associated with higher labor costs [6]. Thus, even if organizations desire to use their data to drive clinical or operational decisions, it is often not feasible without significant resources devoted to this process.

Even when the availability of resources is not a barrier and advanced NLP is utilized to extract text-based data from clinical progress notes, there are still challenges to obtaining usable data and developing accurate models [6]. For example, many clinical progress notes contain text and information that can be extremely similar or the same as a result of providers copying-and-pasting to save time [4,7,8]. As a result, data extracted from these notes can be highly skewed and may result in overfitted models with limited generalizability [6,7]. In other cases, content in clinical progress notes may significantly vary, especially with regard to behavioral health information [9]. Whereas some providers include detailed documentation of factors that may be likely to impact treatment outcomes, such as the type and duration of interventions provided, referrals to other providers, or changes in clients’ symptoms over the course of therapy, other providers may only include the minimal required documentation into their progress notes. Overall, clinical progress notes are highly prone to documentation errors that can lead to missing or incorrect treatment information and the additional burden of extracting usable data. Consequently, it is critical to identify and use approaches that improve the data capture process without adding a significant burden to providers.

One strategy to improve the data capture process is by developing tailored flow sheets within EHRs that are specific to the needs of each clinic and type of encounter. These flow sheets can then be implemented at virtually all levels of patient interaction to allow for the collection of relevant, usable data whenever there is contact with the patient. For example, postencounter forms (PEFs) are flow sheets that can be attached to each clinical encounter and enable clinicians to quickly, accurately, and completely document relevant treatment information that can be easily extracted for analytic purposes. For behavioral health, this may include information about interventions used during sessions, minutes spent providing the interventions, clinical severity and progress, referrals provided, or termination status. The adaptability and convenience of PEFs allow organizations and providers to easily and accurately capture information relevant to their specific needs.

In this case report, we documented the development and implementation of the PEF system in the Road Home Program at Rush University Medical Center. Specifically, we detailed the process of developing the PEF content and system, demonstrated the types and volume of data that can be captured and easily extracted using this system, and described the implementation of the system.

## Case Description

The PEF system was first developed and implemented at the Road Home Program within the Department of Psychiatry at Rush University Medical Center in Chicago, IL in response to a need to easily document and evaluate program use and effectiveness and be able to provide detailed program information to its funders. Later, this system was disseminated to other departments within the academic medical center.

## Methods

### Development of the Postencounter Form

Members from various teams worked together during the PEF system development process to identify their unique data needs and note specific limitations. Teams included *clinical providers* who deliver services and were responsible for entering session data, members from the *knowledge management team* that combine data from multiple sources including the EHR and online survey tools, members from the *data team* who are responsible for cleaning and auditing the data to ensure its accuracy, and members from the *research team* who use data for descriptive and predictive analytics to generate insights that can help improve clinical care. The goal was to develop a comprehensive yet efficient system that enabled the collection of all relevant data in 90 seconds or less per patient encounter to prevent an excessive burden on clinicians who have limited time for documentation. Moreover, the PEF system needed to be *adaptable* since program needs would change as it expanded. It was critical for the system to be *easy-to-use* for all involved parties so that the tool could be quickly learned and errors could be minimized or easily detected and corrected. The PEF system also needed to be *accessible* and enable the immediate extraction of data for analytic purposes. In addition, as described earlier, it was important for the system to be *cost-effective* as the cost of implementing more advanced approaches involving NLP can be prohibitive. The clinical data that were determined to be important to capture in the PEF for the Road Home Program along with a brief description of their purposes are shown in Table 1.

**Figure 1:**
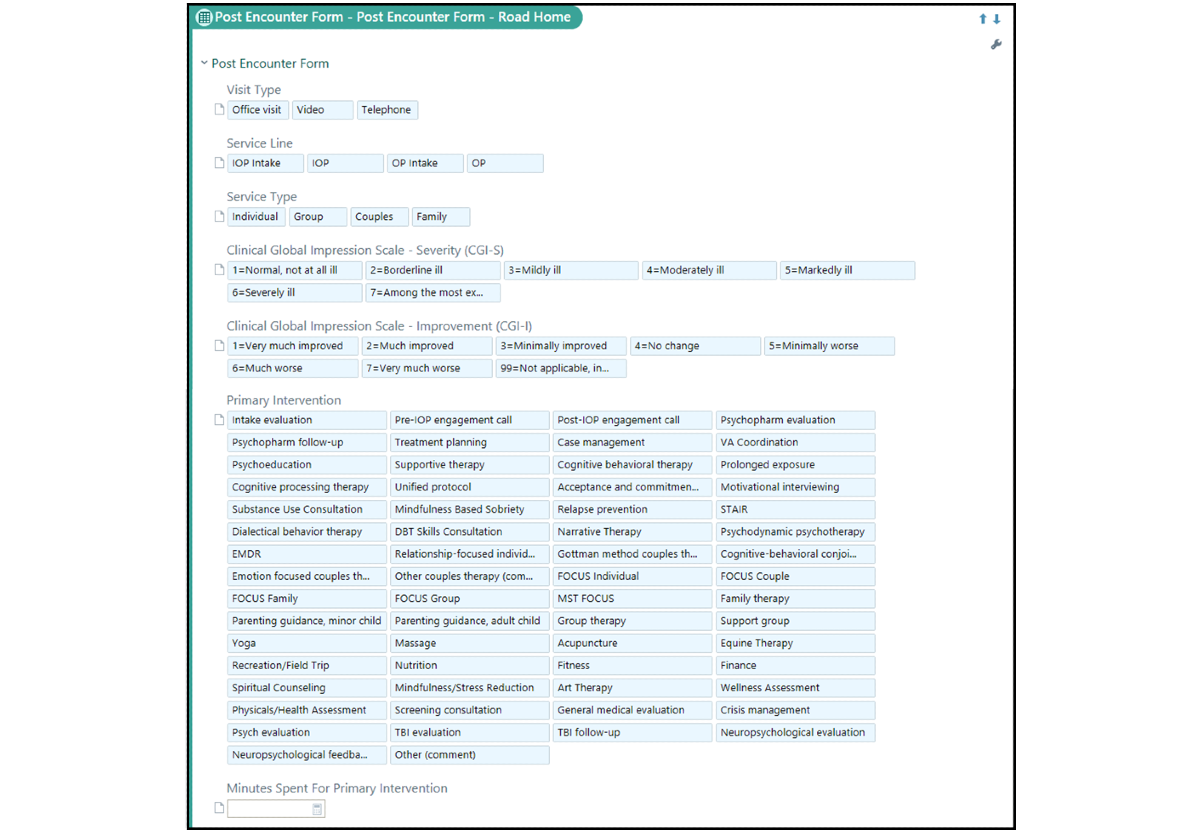
Sample Road Home Program post-encounter form. IOP: intensive out patient; OP: out patient; TBI: traumatic brain injury; VA: Department of Veterans Affairs; STAIR: Skills Training in Affective and Interpersonal Regulation; DBT: dialectical behavior therapy; EMDR: eye-movement desensitization and reprocessing; FOCUS: Families OverComing Under Stress; MST: Military Sexual Trauma.

Figure 1 shows the Road Home Program PEF. The specific fields of the PEF may differ based on a clinic’s needs.

**Table 1:** Road Home Program postencounter form fields with descriptions

Postencounter form field	Description
Visit type	Specify whether encounters took place in person, over the phone, or through video
Service line	Specify whether service provided was an intake vs regular appointment and whether the service was part of the outpatient or 3-week intensive treatment program
Service type	Specify whether services were provided for individuals, groups, families, or couples
Clinical Global Impression Scale (severity)	Validated clinician-rated scale to indicate a patient’s symptom severity level
Clinical Global Impression Scale (improvement)	Validated clinician-rated scale to indicate how much a patient has improved
Primary intervention	Specify the type of intervention delivered
Minutes spent for primary intervention	Specify the number of minutes for which the intervention was delivered
Secondary intervention	Specify the type of any secondary intervention delivered, when applicable
Minutes spent for secondary intervention	Specify the number of minutes for which the secondary intervention was delivered
Referral given	Specify the type of referral that was made during the session
Referral target	Specify for whom the referral was made
Referral reason	Specify the reason the referral was made
Termination	Specify whether termination took place during the session
Termination date	Specify the date of the termination
Leave reason	Specify the reason for leaving or terminating care

### Implementation into the Road Home Program

**Figure 2:**
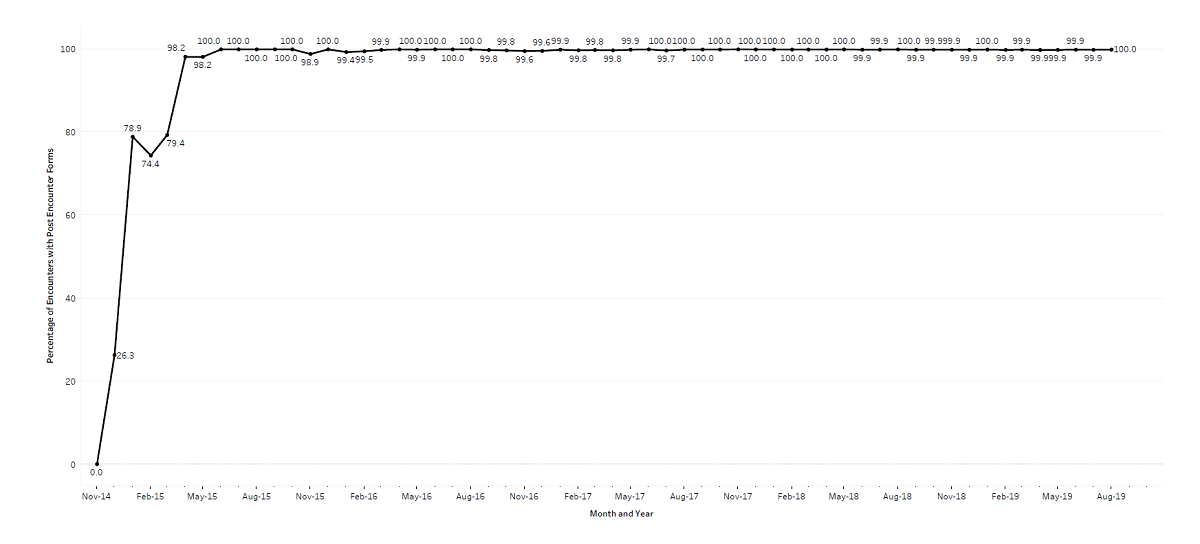
Percentage of Road Home Program encounters with post-encounter forms from November 2014 through September 2019.

Following the development of the PEF system, which involved evaluating various prototypes, the team implemented it into the Road Home Program clinical flow. All Road Home Program staff were required to attend a 1-hour training session where the purpose of the PEF was discussed. A demonstration was given on how to complete the form and questions about its use were answered. The difficulty of extracting data from clinical progress notes was specifically highlighted to help staff understand the importance of collecting data in easily extractable ways, even if this process added up to 90 seconds per encounter. Following the initial training, the team periodically met with select providers to ensure that everyone was aligned with regard to how the PEFs were completed, for example to ensure that providers would all code a cognitive behavioral intervention in the same way rather than using another, noncognitive behavioral intervention code. Several weeks following the training, the team met with the staff again to demonstrate the usefulness of PEFs by providing basic descriptive information about encounters that were already completed using PEFs. The descriptive information focused on the number of minutes spent delivering interventions such as cognitive behavioral therapy, and the types of referrals that had been made and tracked since the implementation of the PEF. The intention behind this process was to reinforce providers’ use of the PEF. Clinicians quickly began to use the PEF following its implementation (Figure 2). In only 5 months, 98.2% (109/111) of all Road Home Program clinical encounters included a PEF. The rate of use has remained extremely high since the PEF system was implemented; on average, only 0.08% (260/323,026) of all encounters had a missing PEF since May 2015. Between December 2015, and August 2019, a total of 40,889 PEFs have been completed by Road Home Program providers, capturing more than 325,188 unique data points.

To facilitate the implementation, the team met with providers who tended to underutilize the PEF system to identify and resolve potential barriers. The biggest barrier to using the PEF was providers not being able to locate it in the EHR, which lead to the creation of a specific instructional guide on where to locate it. The team continued to meet with providers as needed when they encountered issues with locating or completing the form following the initial implementation and will continue to do so as needed. The team also continued to present descriptive and advanced analytical insights about the program to providers to further reinforce the utility and importance of the PEF system.

## Discussion

In this case study we described the development and implementation of the PEF system, which can be used to quickly and accurately capture clinically relevant data in a comprehensive yet efficient, adaptable, easy-to-use, accessible, and cost-effective way. Given the challenges associated with manual extraction of clinical progress note content or the use of more advanced and costly processes such as NLP, it was important to identify ways that clinical data could be easily captured and made immediately usable for analytic purposes. This study demonstrated the PEF system built in Epic; however, it can be built in to most modern EHR systems with minimal effort, as it is based on simple flow sheets that are attached to each encounter. Moreover, the PEF system is highly adaptable to the unique needs of any department or clinic. It is also flexible enough that its content can be changed as priorities or needs shift.

The implementation of the PEF system at the Road Home Program demonstrated the importance of collaboration between members and different teams. A common challenge to implementing new systems is that direction is given top-down with little input from individuals who are expected to use newly developed tools. In this case, members from a wide range of teams worked together to develop an end product that met the needs of everyone involved. Moreover, the development team worked closely with providers during the implementation phase and ensured that providers understood why the system was developed and the benefits of the system compared to existing practices (ie, clinical progress notes alone). The development team then reinforced the use of the newly developed PEF system by repeatedly demonstrating to providers what data was being generated and how it can be used to inform clinical or operational decisions.

It is important to note that the use of flow sheets is not limited to PEFs, as flow sheets can be used to capture all types of data. Since the successful implementation of the PEF system, the Road Home Program has implemented this system for most other forms of data collection such as demographic information during an intake evaluation, medical information, or clinician-administered assessment data. Anecdotally, providers continue to remark about the ease of data collection. The data captured using the PEF system have been critical in providing data for the development of predictive models that have led to the improvement of clinical operations [10].

This case study has several limitations that should be noted. First, this case study focused on a single program within one academic medical center. Additional research needs to be conducted to determine whether the PEF is a viable option for other departments and medical systems. Second, the implementation of the PEF system was not randomized. Thus, it is impossible to determine in what ways the close collaboration between the development team and providers as well as the stepwise approach affected the ultimate uptake. Last, the PEF system was not directly compared to approaches using NLP. Thus, it cannot be determined whether the PEF system is more or less accurate, labor-intensive, or costly than NLP-based approaches.

Despite the aforementioned challenges, this case study highlighted how useful the PEF system can be for quickly and accurately capturing data without increasing provider burden or requiring significant time or funding to extract clinical data. Future research should closely examine the implementation process to determine the most effective way of rolling out new tools such as the PEF in a variety of health settings.

## Abbreviations

EHR: electronic health record
NLP: natural language processing
PEF: postencounter form.
